# DEspR^high^ neutrophils are associated with critical illness in COVID-19

**DOI:** 10.1038/s41598-021-01943-7

**Published:** 2021-11-17

**Authors:** Joanne T. deKay, Ivette F. Emery, Jonathan Rud, Ashley Eldridge, Christine Lord, David J. Gagnon, Teresa L. May, Victoria L. M. Herrera, Nelson Ruiz-Opazo, Richard R. Riker, Douglas B. Sawyer, Sergey Ryzhov, David B. Seder

**Affiliations:** 1 Maine Medical Center Research Institute, 81 Research Drive, Scarborough, ME 04074 USA; 2 Department of Critical Care Services, Maine Medical Center, 22 Bramhall St, Portland, ME 04105 USA; 3 Whitaker Cardiovascular Institute and Department of Medicine, Boston University School of Medicine, Boston, MA USA; 4 Tufts University School of Medicine, Boston, MA USA

**Keywords:** Immunology, Diseases

## Abstract

SARS-CoV-2 infection results in a spectrum of outcomes from no symptoms to widely varying degrees of illness to death. A better understanding of the immune response to SARS-CoV-2 infection and subsequent, often excessive, inflammation may inform treatment decisions and reveal opportunities for therapy. We studied immune cell subpopulations and their associations with clinical parameters in a cohort of 26 patients with COVID-19. Following informed consent, we collected blood samples from hospitalized patients with COVID-19 within 72 h of admission. Flow cytometry was used to analyze white blood cell subpopulations. Plasma levels of cytokines and chemokines were measured using ELISA. Neutrophils undergoing neutrophil extracellular traps (NET) formation were evaluated in blood smears. We examined the immunophenotype of patients with COVID-19 in comparison to that of SARS-CoV-2 negative controls. A novel subset of pro-inflammatory neutrophils expressing a high level of dual endothelin-1 and VEGF signal peptide-activated receptor (DEspR) at the cell surface was found to be associated with elevated circulating CCL23, increased NETosis, and critical-severity COVID-19 illness. The potential to target this subpopulation of neutrophils to reduce secondary tissue damage caused by SARS-CoV-2 infection warrants further investigation.

## Introduction

Overactivation of the immune system is thought to play a role in the pathophysiology of severe acute respiratory syndrome coronavirus 2 (SARS-CoV-2) infection^1–3^; the mainstays of therapy for severe disease include reduction in viral load, corticosteroids, and IL-1 and IL-6 inhibitory treatments, which act to suppress the immune response^4,5^. Proposed mechanisms of immune dysfunction in severe COVID-19 include T cell deficiencies and dysregulation of lymphocyte response^6^, excessive inflammation^7^, and aberrant complement activation^8,9^.

Increased numbers of circulating neutrophils are typically seen during COVID-19^10^. High neutrophils in combination with a low number of circulating lymphocytes predict a poor outcome^11^. The increased number of immature CD10 negative neutrophils has been described and also correlates with the severity of COVID-19^12^. Infiltration of neutrophils in the lungs has also been documented in patients infected with SARS-Cov-2, specifically in those developing acute respiratory distress syndrome^13^. However, not only the number but also the functional status of neutrophils is different in COVID-19 subjects^14^. Factors contributing to the shift in their functional properties include mobilization of immature cells from the bone marrow and activation of neutrophils in the circulation, resulting in increased heterogeneity in the pool of circulating neutrophils.

An increased number of immature CD10 negative neutrophils has been described and correlates with the severity of COVID-19^12^. Blood accumulation of abnormal neutrophils occurs^15,16^ and likely indicates the activation of immature neutrophils with pro-inflammatory factors, released during a systemic inflammatory response^17^. While mature and properly activated neutrophils are protective, aberrant activation of immature neutrophils may contribute to excessive inflammatory and secondary tissue damage. The development of targeted therapeutic approaches to prevent unnecessary immune cell-mediated tissue damage requires further investigation of neutrophil heterogeneity^18^.

The Dual Endothelin-1 and VEGF signal peptide-activated Receptor (DEspR) is a single transmembrane receptor coupled to a Ca^2+^-mobilizing transduction pathway^19^. DEspR is essential for embryonic angiogenesis and neuroepithelial development. DEspR deficiency is associated with embryonic lethality^20^. Endothelin-1 is an endogenous ligand that binds DEspR within the range of low nanomolar concentrations^20^. Both endothelin-1 and Ca^2+^ intracellular signaling play an important role in activating innate immunity^21,22^. However, the role of DEspR in the regulation of myeloid cells, and specifically in neutrophils, has not been described.

The goals of this study were to characterize the expression of DEspR in subpopulations of immune cells in patients with COVID-19 and healthy donors and to determine the associations between the number of immune cells expressing a high level of DEspR (DEspR^high^), inflammatory factors, and the severity and clinical features of COVID-19.

## Results

### Study subjects

A convenience sample of 26 patients with COVID-19 and 12 control samples were analyzed. Among our COVID-19 study group, 6 patients (23%) were characterized by mild illness, 9 subjects (35%) had a severe illness, and 11 subjects (42%) were classified with a critical illness.

There were no differences in age or sex between study subjects and controls. A comparison of the demographics and clinical characteristics of COVID-19 and control subjects is provided in Table 1.

**Table 1:** Characteristics of control subjects and subjects with COVID-19.

Characteristic	Control subjects (n = 12)	Subjects with COVID-19 (n = 26)
Age, mean ± SD	67 ± 9.6	63.7 ± 15.4
Male, n (%)	8 (66.7)	16 (61.5)
Race, (%)	White (100)	White (100)
BMI, mean ± SD	28.2 ± 5	32 ± 7
COVID severity*, # (%)		
Mild, severe, critical		6, 9, 11 (23, 35, 42)
Estimated FiO2 (%), median (IQR)		36, (29, 75)
Days since symptom onset, mean ± SD		8.0 ± 4.8
Antibiotics, # (%)		17 (65.4)
Corticosteroids, # (%)		21 (80.8)
Hemodialysis, # (%)		3 (11.5)
Thrombosis, # (%)		6 (23.1)
Hospital LOS, median (IQR)		8 (4, 14)
Discharge survival, # (%)		21 (80.8)

*IQR* interquartile range, *FiO2* fraction of inspired oxygen, *LOS* length of stay.

*Mild: no need for supplemental oxygen; Severe: supplemental oxygen required; Critical: critically ill with respiratory failure.

### The number of DEspR^high^ cells is increased in patients with COVID-19 and correlates to disease severity

**Figure 1:**
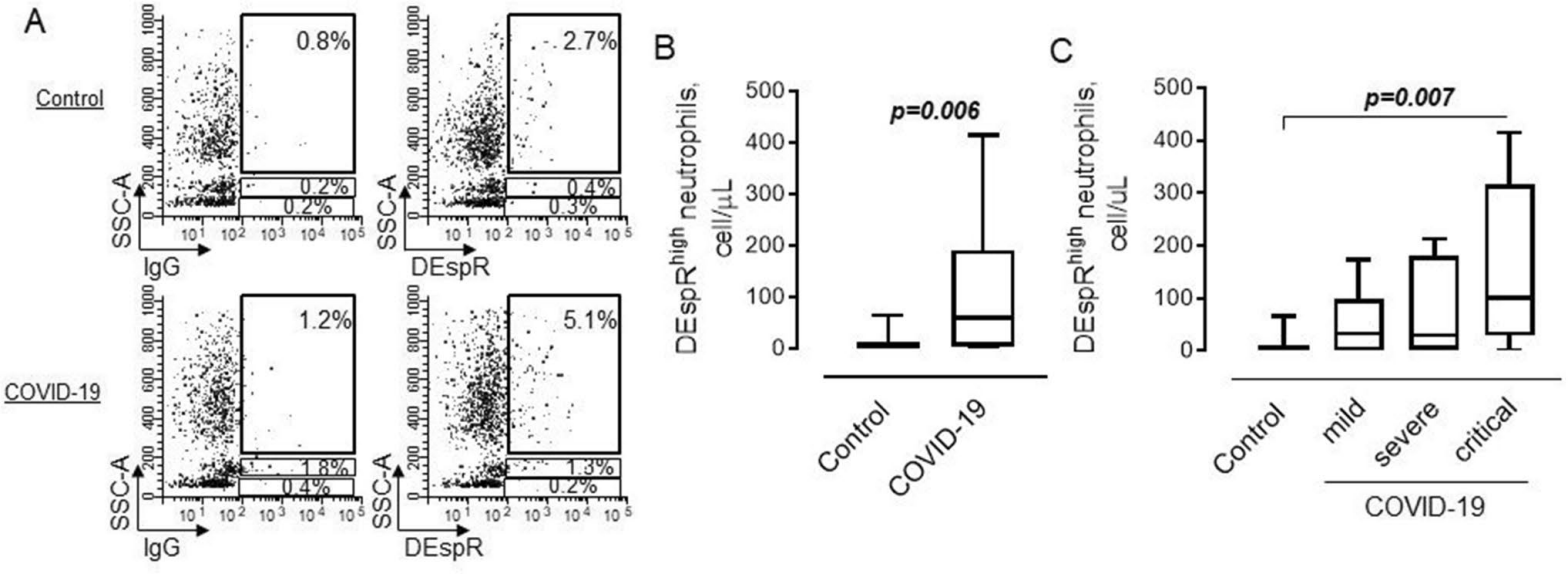
Flow cytometric strategy to determine DEspR^high^ cells in the peripheral circulation. Freshly obtained blood cells were analyzed after erythrocytes lysis. (**A**) Flow cytometric plots showing gating strategy for cells expressing a high level of cell surface DEspR in control (upper) and COVID-19 (lower) subjects. (**B**) Graphical representation of data from flow cytometric analysis on the number of DEspR^high^/SSC^high^ neutrophils in control (*n* = 12) and COVID-19 (*n* = 26) patients; Mann–Whitney test. (**C**) The number of DEspR^high^/SSC^high^ neutrophils in control subjects and patients with mild (*n* = 6), severe (*n* = 9), or critical (*n* = 11) COVID-19. Kruskal–Wallis test, Dunn’s multiple comparisons test; *P* value is shown.

To determine the cell surface expression of DEspR on circulating immune cells, we used flow cytometric analysis. Gates for cells expressing a high level of DEspR within major subpopulations of SSC^high^ neutrophils, SSC^intermediate^ monocytes, and SSC^low^ lymphocytes were set up using isotype IgG’s as shown in Fig. 1A. The majority of cells with a high level of DEspR at their surface are characterized by high intracellular granularity (SSC^high^), which allowed us to identify these cells as neutrophils. These cells were found in both healthy subjects and COVID-19 patients. However, the number of DEspR^high^ neutrophils was increased in COVID-19 patients compared to controls (Fig. 1B). The analysis also revealed the accumulation of DEspR^high^ cells in the blood of critically ill COVID-19 patients (Fig. 1C).

### DEspR^high^ neutrophils express CD10 and elevated levels of CD14

**Figure 2:**
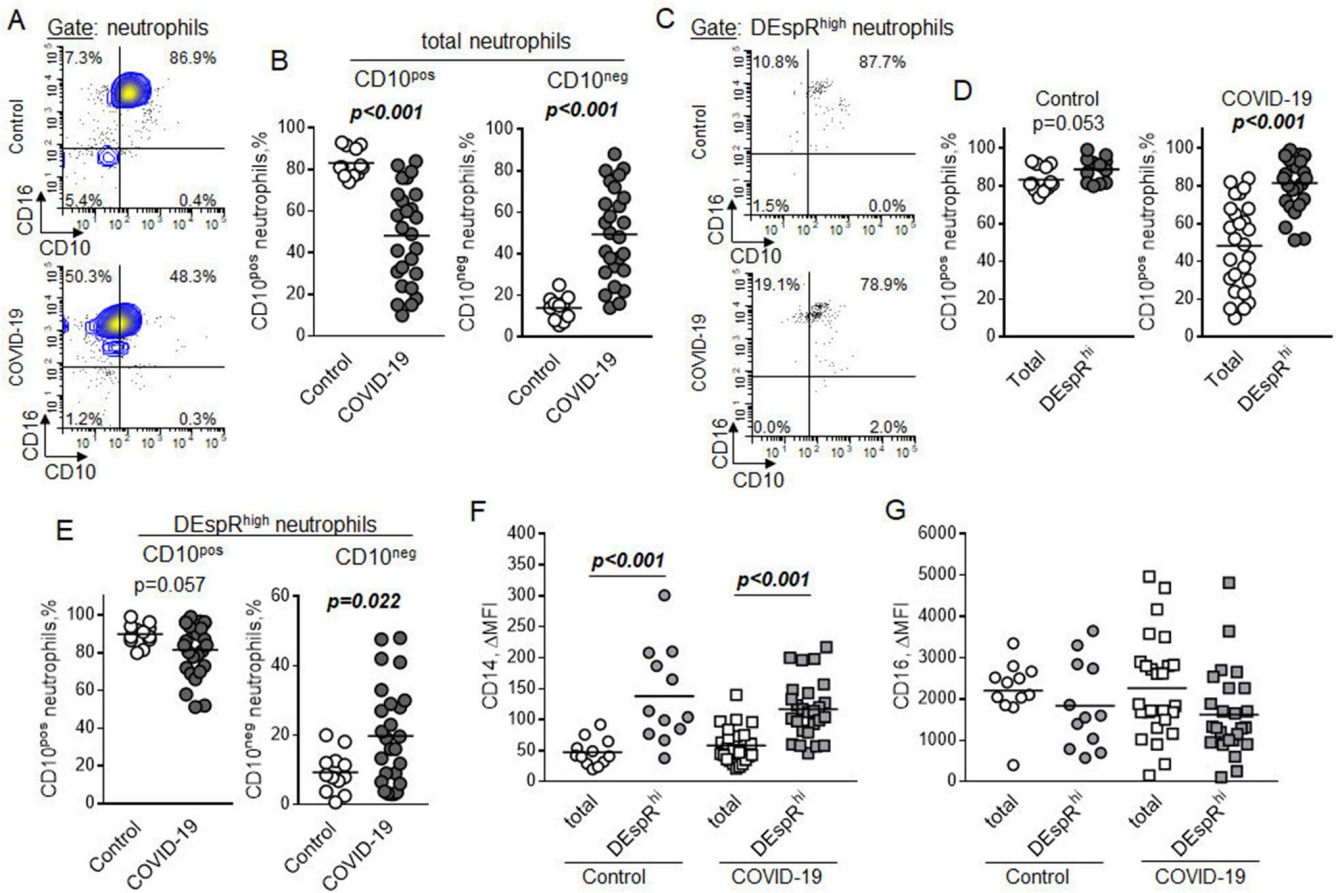
Expression of CD10, CD14, and CD16 on DEspR^high^ neutrophils in control subjects and COVID-19 patients. The expression of cell surface markers was determined in the subpopulation of peripheral blood SSC^high^ neutrophils (*total neutrophils*) or a subset of neutrophils with high expression of DEspR (*DEspR*^*high*^* neutrophils*) in control (*n* = 12) and COVID-19 (*n* = 26) subjects. (**A**) Representative flow cytometric plots showing expression of CD10 on total neutrophils in control (upper) and COVID-19 (lower) subjects. (**B**) Number of CD10 positive (left) and CD10 negative neutrophils. Mann–Whitney test. (**C**) Flow cytometric plots demonstrating expression of CD10 on DEspR^high^ neutrophils in control (upper) and COVID-19 (lower) subjects. (**D**) Percentage of CD10 positive cells in a subpopulation of total neutrophils (Total) or a subset of DEspR^high^ neutrophils in control (left) and COVID-19 (right) subjects. Mann–Whitney test. (**E**) Percentage of CD10 positive (left) and CD10 negative (right) neutrophils in control subjects and COVID-19 patients. Mann–Whitney test. (**F,G**) Expression of (**F**) CD14 and (**G**) CD16 on total and DEspR^high^ neutrophils in control and COVID-19 subjects. The expression is represented by the mean fluorescence intensity that corresponds to the level of cell surface CD14 and CD16. ΔMFI was calculated by subtracting the mean fluorescence intensity of isotype controls from the mean fluorescent intensity of specific antibodies. Two-way ANOVA with Tukey multiple comparisons test; *P* values are indicated.

To better characterize the antigenic phenotype of cells with high expression of DEspR, we performed the analysis of markers associated with neutrophils maturation and activation, CD10 and CD16, in the subpopulation of total neutrophils and subset of DEspR^high^ neutrophils. High level expression of CD16 was found on the surface of more than ninety percent of cells in the total subpopulation of SSC^high^ neutrophils in both control subjects and patients with COVID-19 (Fig. 2A). The majority of neutrophils from the control subjects were also characterized by the expression of CD10, whereas neutrophils from patients with COVID-19 did not express CD10, a marker associated with neutrophils maturation^23^, on their surfaces (Fig. 2B).

We also determined the percentage of CD16 and CD10 positive cells in the subset of DEspR^high^ neutrophils. Similar to the subpopulation of total neutrophils, the majority of DEspR^high^ neutrophils expressed CD16 (Fig. 2C). Although not statistically significant, this subset of DEspR^high^ neutrophils demonstrated a trend toward a higher percentage of CD10 expressing cells compared to total neutrophils in control subjects (Fig. 2D). The high percentage of neutrophils that express CD10 within the subset of DEspR^high^ neutrophils indicated their more mature state compared to total neutrophils in COVID-19 patients. There was a difference in the maturation state of DEspR^high^ neutrophils between control and COVID-19 subjects. A trend toward a lower percentage of CD10 positive neutrophils and a significantly higher percentage of CD10 negative neutrophils were found in the subset of DEspR^high^ neutrophils in patients with COVID-19 compared to control subjects (Fig. 2E).

CD14 is associated with pro-inflammatory activation of neutrophils^24,25^. DEspR^high^ neutrophils were characterized by significantly higher expression of CD14 compared to total neutrophils in both groups of study subjects (Fig. 2F). No differences were found in the level of CD14 on the surface of total and DEspR^high^ neutrophils between control and COVID-19. There was no difference in the cell surface expression of CD16 between total and DEspR^high^ neutrophils or neutrophils in the groups of control subjects and patients with COVID-19 (Fig. 2G).

### Lack of association between the number of DEspR^high^ neutrophils and lymphocyte count in COVID-19

**Figure 3:**
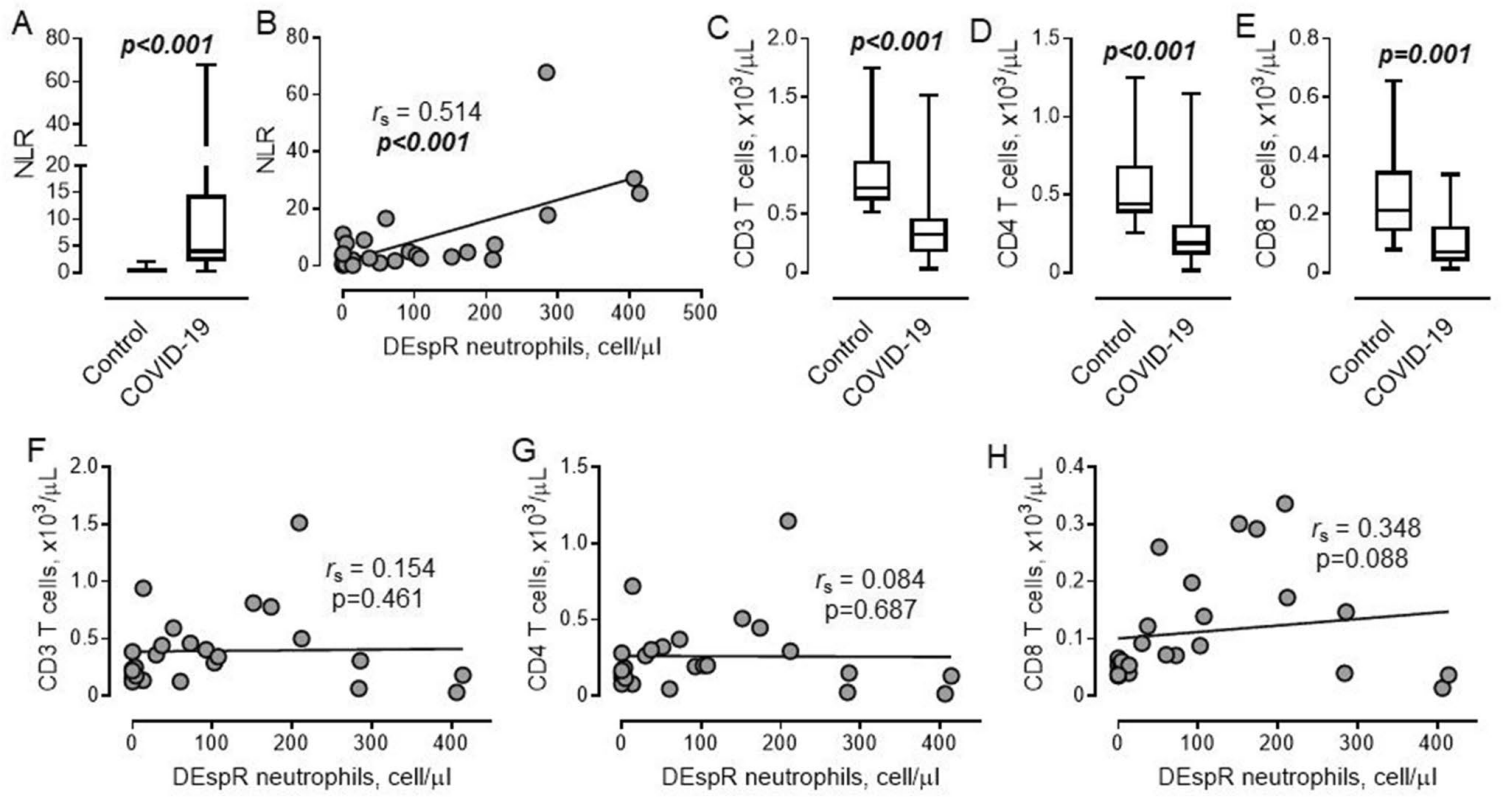
High neutrophil to lymphocyte ratio is associated with an increased number of DEspR^high^ neutrophils. (**A**) Neutrophil–lymphocyte ratio (*NLR*) in control (*n* = 12) and COVID-19 (*n* = 26) subjects. Mann–Whitney test. (**B**) Relationship between NLR and number of DEspR^high^ neutrophils. Spearman correlation coefficient is indicated. (**C–E**) Number of (**C**) CD3 T lymphocytes, (**D**) CD3/CD4 and (**E**) CD3/CD8 T cells in control and COVID-19 subjects. Mann–Whitney test. (**F–H**) Association between the number of DEspR^high^ neutrophils and (**F**) total T lymphocytes, (**G**) CD3/CD4, and (**H**) CD3/CD8 T cells. Spearman correlation coefficients and *P* values are indicated.

Our data demonstrate that the neutrophil-to-lymphocyte ratio (NLR) is significantly increased in patients with COVID-19 compared to control (Fig. 3A). We also found a positive correlation between the number of DEspR^high^ neutrophils and NLR in COVID-19 (Fig. 3B). Because neutrophils possess immunosuppressive properties^26^, we separately examined the potential relationship between both DEspR^high^ neutrophils and lymphocytes in the circulation.

The number of CD3 lymphocytes and major subsets of CD4 and CD8 T cells were significantly decreased in COVID-19 compared to control subjects, indicating enhanced systemic immunosuppression (Fig. 3C–E). However, no associations were found between the number of DEspR^high^ neutrophils and the total number of T cells and CD4 lymphocytes (Fig. 3F,G). Although not statistically significant, a trend toward a weak positive association was found between DEspR^high^ neutrophils and CD8 T cells in patients with COVID-19 (Fig. 3H).

### Circulating CCL23 is increased in COVID-19

**Figure 4:**
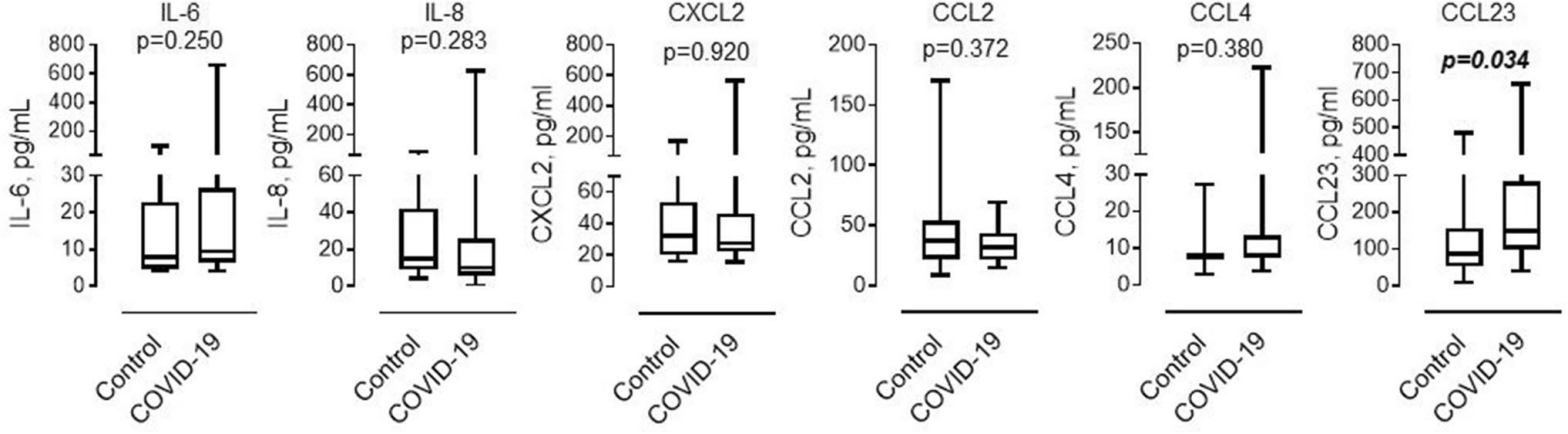
Level of circulating pro-inflammatory factors in control and COVID-19 subjects. Levels of circulating cytokines and chemokines were determined in platelet-free plasma in groups of control subjects (*n* = 12) and COVID-19 patients (*n* = 25). One patient with extracorporeal membrane oxygenation support was excluded from the analysis due to the potential effect of cytokine adsorption during ECMO therapy. Mann–Whitney test.

In parallel with the flow cytometric analysis of DEspR^high^ neutrophils, we determined the level of cytokines and chemokines that contribute to the activation and recruitment of neutrophils. As shown in Fig. 4, the level of CCL23 was significantly increased in patients with COVID-19 compared to control subjects. No differences were found between the two groups in the levels of IL-6, IL-8, CXCL2, CCL2, or CCL4.

### DEspR^high^ neutrophils are associated with increased CCL23

**Figure 5:**
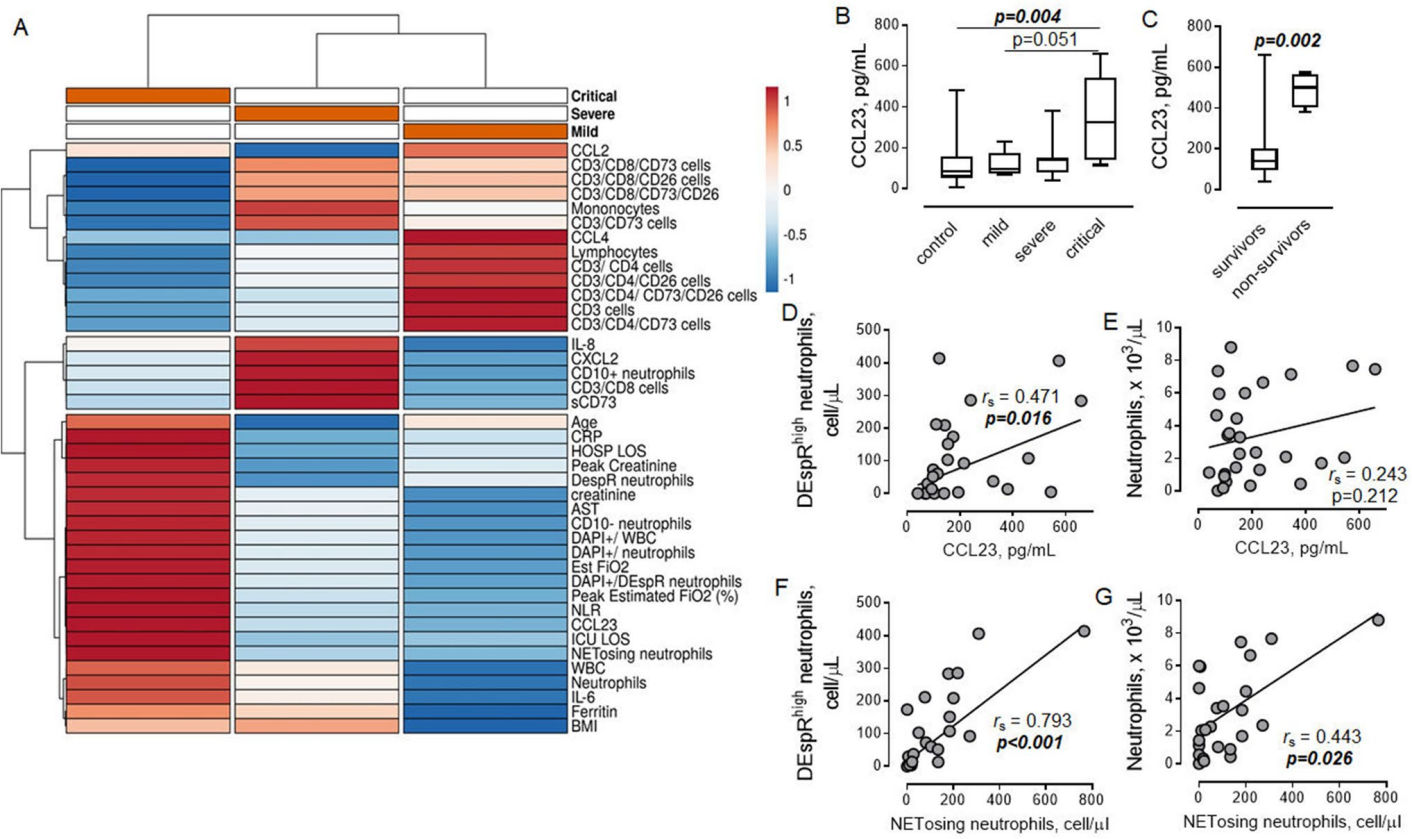
Association between DEspR^high^ neutrophils and CCL23. (**A**) Heat map associated with hierarchical clustering analysis of tested parameters in groups of mild (*n* = 6), severe (*n* = 9), and critical (*n* = 10) COVID-19 patients obtained using ClustVis 2.0. Columns with similar annotations are collapsed by taking the median inside each group. Rows are centered; unit variance scaling is applied to rows. Both rows and columns are clustered using correlation distance and average linkage. Color keys show the relative strength of the signal in each cluster group. Annotations on top of the heatmap show clustering of the samples. *AST* aspartate aminotransferase, *BMI* body mass index, *CRP* C-reactive protein, *DAPI*+ 4′,6-diamidino-2-phenylindole viability dye positive dead cells, *Est FiO2* estimated fraction of inspired oxygen, *HOSP LOS* hospital length of stay, *ICU LOS* intensive care unit length of stay, *sCD73* soluble CD73. (**B**) Association between the level of circulating CCL23 and COVID-19 severity. Spearman correlation coefficient and *P* value are indicated. (**C**) The level of CCL23 in COVID-19 survivors (*n* = 21) and non-survivors (*n* = 4). Mann–Whitney test. (**D,E**) Associations between the level of CCL23 and number of (**D**) DEspR^high^ neutrophils and (**E**) total neutrophils in COVID-19 patients. (**F,G**) Relationships between the number of NETosing neutrophils and number of (**F**) DEspR^high^ neutrophils and (**G**) total neutrophils in COVID-19 patients. (**D–G**) Spearman correlation coefficients and *P* values are indicated.

To determine the potential relationship between DEspR^high^ neutrophils and other measured parameters in relation to disease severity, a hierarchical clustering analysis in a group of patients with COVID-19 was performed. As shown in Fig. 5A, the majority of lymphocyte subpopulations, CCL2, and CCL4, were clustered with mild illness. As expected, DespR^high^ neutrophils were clustered within the group of critically ill patients with COVID-19. In addition, we found that CCL23 is also associated with a critical illness (Fig. 5B), and that CCL23 is significantly increased in non-survivors (Fig. 5C).

A positive correlation was found between the number of DEspR^high^ neutrophils, but not the total number of neutrophils, and the level of CCL23 (Fig. 5D,E), indicating there may be a direct relationship between these two factors in the development of critical COVID-19. In addition, our analysis revealed positive correlations between the number of NETosing neutrophils and the number of both DEspR^high^ and the total subpopulation of neutrophils (Fig. 5F,G).

## Discussion

This study demonstrates the presence of a newly defined subset of neutrophils characterized by high cell surface expression of DEspR in human blood. The number of these DEspR^high^ neutrophils is increased in patients with COVID-19 and correlates with disease severity. Elevated expression of CD10 and CD14, markers of maturation and activation, may indicate the high pro-inflammatory potential of these neutrophils. An association was seen between the number of DEspR^high^ neutrophils, NETosing neutrophils, the blood plasma level of CCL23, and critical illness in COVID-19.

Accumulating evidence suggests that neutrophils are major players in the development of hyperinflammation associated with poor outcomes in patients with SARS-CoV-2 infection^27^. Neutrophils may contribute to disease progression via several mechanisms, including immunosuppression^28^, production of pro-inflammatory factors^29^, and activation of coagulation and thrombosis^30^. Recently it has been recognized that circulating neutrophils are a heterogeneous population of cells^31^. A subset of mature CD10-expressing neutrophils is characterized by the ability to suppress T lymphocytes^23^, which may contribute to systemic immunosuppression and prevent effective viral clearance^32^. It has also been shown that decreased lymphocyte count is associated with severe and critical conditions in COVID-19^33,34^. Clustering analysis of data generated in our study demonstrated an association between immunosuppressive factors such as soluble CD73^35^, CD10 positive neutrophils^23^ and IL-8^36^, and severe COVID-19. We also found that DEspR^high^ neutrophils are characterized by the high expression of CD10. Our analysis revealed a positive correlation between DEspR^high^ neutrophils and NLR and a significantly decreased number of lymphocytes in patients with COVID-19. In addition, we found differential clustering of DEspR^high^ neutrophils to the group of critically ill patients compared to lymphocyte clustering in the group of patients with mild COVID-19 disease, pointing toward potential immunosuppressive properties of neutrophils expressing a high level of DEspR. No correlations, however, were found between DEspR^high^ neutrophils and different subsets of circulating CD3 lymphocytes in the current study. Thus, our data indicate that DEspR^high^ neutrophils are unlikely to be involved in the regulation of systemic lymphocyte trafficking or suppression. Further studies are warranted to determine the direct effect of DEspR^high^ neutrophils on lymphocyte activation locally in inflamed tissue.

While the majority of DEspR^high^ neutrophils express CD10 in both study groups, the percent of CD10 negative neutrophils within the DEspR^high^ subset is higher in patients with COVID-19 compared to control, reflecting the increased mobilization of immature neutrophils from the bone marrow in COVID-19. This also indicates that the upregulation of DEspR expression on neutrophils is not dependent on their maturation and is initiated in the peripheral circulation. This should result in their accumulation in the blood, however, the overall percent of DEspR^high^ neutrophils is low. One possible explanation is related to the egress of DEspR^high^ neutrophils out of the bloodstream due to their migration into tissues. It has been shown previously that endothelin-1, a DEspR ligand, enhances the adhesion of neutrophils to endothelial cells^37–39^ and promotes the recruitment of neutrophils into inflamed tissue^40–42^.

Another important observation from this study is related to the high expression of CD14 on DEspR^high^ neutrophils. CD14 is a coreceptor for toll-like receptors^43,44^. Upon pro-inflammatory activation, the cell surface expression of CD14 is increased^45^ and promotes synthesis and secretion of pro-inflammatory factors, such as tumor necrosis factor-alpha^46^. CD14 is involved in the activation of both monocytes and neutrophils in vasculitis^47,48^, one of the common cardiovascular complications in COVID-19^49^. While high expression of CD14 is associated with activation of neutrophils, our data demonstrate that DEspR^high^ neutrophils also express a high level of CD16 comparable to the level of expression found on most neutrophils in the blood. A high level of CD16 expression is usually found on resting, non-activated neutrophils^50^. Apoptotic neutrophils, subsets of immature or activated neutrophils, are characterized by a significant reduction in the cell surface expression of CD16^50^. The high levels of CD14 expression on DEspR^high^ neutrophils may indicate their enhanced pro-inflammatory potential compared to the overall neutrophil population.

Chemokines are critical factors in the recruitment of immune cells to the area of inflammation^51^. Our study demonstrated elevated levels of circulating CCL23 in patients with COVID-19. CCL23 is a potent chemoattractant for the peripheral blood mononuclear cells, including monocytes, dendritic cells, and resting lymphocytes^52,53^. Both monocytes and neutrophils produce CCL23 in response to a variety of toll-like receptor ligands^54^. However, in contrast to the majority of cytokines and chemokines secreted at a significantly higher level from monocytes, neutrophils produce high levels of CCL23. Furthermore, toll-like receptor ligands induce early neutrophils response compared to delayed upregulation and secretion of CCL23 in monocytes^54^. Neutrophils may represent a major source of CCL23 considering the significant increase in the number of neutrophils during the systemic inflammatory response. The expression of CCL23 by brain tissue-infiltrating neutrophils after a stroke has been reported^55^. Our analysis revealed a positive correlation between the number of DEspR^high^ neutrophils, but not total neutrophils, and the level of circulating CCL23 in patients with COVID-19. Both DEspR^high^ neutrophils and CCL23 were significantly increased in critically ill patients with COVID-19. Given a high expression of CD14 that promotes toll-like receptor signaling, it is plausible that DEspR^high^ neutrophils produce CCL23 to further promote inflammation. This is supported by our data showing that a high level of CCL23 is associated with poor outcomes in patients with COVID-19. However, further studies are required to determine the potential role of DEspR^high^ neutrophils in CCL23 secretion.

Neutrophil extracellular traps (NET) propagate inflammation and microvascular thrombosis in patients with COVID-19^56^. Our data reveal a positive correlation between the number of DEspR^high^ neutrophils and NETosing neutrophils in the peripheral circulation of patients with COVID-19, indicating potential involvement of DEspR in the regulation of NET. NETosing neutrophils may induce the secretion of endothelin-1 from endothelial cells^57^, suggesting the presence of an amplification loop that may further promote both NETosis and inflammatory activation of endothelial cells via mechanisms involving the generation of DEspR^high^ neutrophils.

This study included a broad population of patients with COVID-19, and the sample size of subgroups was small, so important variability of individuals within the categories of mild, moderate, and severe disease may either be lost or exaggerated. Most patients were receiving corticosteroids, which may modulate the cellular immune response and could affect the behavior or activation status of lymphocytes and neutrophils; the effect on DEspR expression in vivo is unknown. Critically ill patients also received sedatives, analgesics, and sometimes neuromuscular blocking agents, all of which may affect the inflammatory response^58,59^. Because the study was conducted over 5 months of intensive COVID-19 research and evolving clinical standards, care of early and later patients may have varied somewhat, potentially affecting the immunoinflammatory response. Nonetheless, this study is among the first to characterize a newly identified subset of DEspR^high^ neutrophils in COVID-19, and ultimately its inclusion of healthy controls and patients with the full spectrum of severity of COVID-19 is a strength. Furthermore, the population studied reflects a “real-world” cohort with corresponding complexity and variability, making the strength of our findings even more relevant and clinically important.

In summary, the systemic inflammatory response in patients with COVID-19 is associated with an accumulation of neutrophils expressing a high level of DEspR at the cell surface. Antigenic immunophenotype identifies DEspR^high^ neutrophils as mature neutrophils with high pro-inflammatory properties. A high number of circulating DEspR^high^ neutrophils is associated with an increased level of CCL23 and NETosing neutrophils in critically ill COVID-19 patients, identifying DEspR^high^ neutrophils as an intermediate biomarker to track in clinical trials of therapies for COVID-19 and a new potential therapeutic target for the prevention of immune cell-driven hyperinflammation.

## Methods

### Patients

The study and our informed consent process were approved by the Maine Medical Center Institutional Review Board. Informed consent was obtained from the patient or their legally authorized representative (LAR) using a secure electronic consent form (to prevent disease transmission), either in-person (with the patient) or by telephone (with the LAR). All methods were performed in accordance with the relevant guidelines and regulations. A convenience sample included hospitalized patients that provided informed consent between July and December 2020 within 72 h of being hospitalized with PCR-confirmed SARS-CoV-2 infection (real-time RT-PCR test, NorDx Laboratories, Portland, ME). We excluded patients under 18 years of age, those representing a vulnerable population, and those with hemoglobin < 8 g/dL.

The severity of COVID-19 disease was defined using the following criteria from the US Center for Disease Control (CDC)^60^, (1) *mild illness* individuals who have any signs and symptoms of COVID-19 (e.g., fever, cough, sore throat, malaise, headache, muscle pain) without shortness of breath, dyspnea, or abnormal chest imaging, (2) *severe illness* individuals who have respiratory frequency > 30 breaths per minute, SpO2 < 94% on room air at sea level (or, for patients with chronic hypoxemia, a decrease from baseline of > 3%), a ratio of arterial partial pressure of oxygen to fraction of inspired oxygen (PaO2/FiO2) < 300 mmHg, or lung infiltrates > 50%, and (3) *critical illness* individuals who have respiratory failure, septic shock, and/or multiple organ dysfunction.

Blood samples were additionally obtained from asymptomatic and SARS-CoV-2 negative subjects to serve as a control population. Pertinent demographic and clinical data were collected from the electronic medical record for all study participants.

### Standards of care

Patients received an evolving standard of care during their hospitalization. This included the antiviral drug remdesivir^61^ and the synthetic adrenocortical glucocorticoid dexamethasone^62^; many also received systemic anticoagulation^63^, and antibiotics for community-acquired pneumonia^64^ until it was determined they did not have a concurrent bacterial infection. Our cohort included a few patients who were asymptomatic but determined to have a SARS-CoV-2 infection on administrative testing, although most had a severe or critical disease. Mechanically ventilated patients received lung-protective ventilation according to international clinical practice guidelines^65^, proning for refractory hypoxemia^66^, and standard evidence-based critical care therapies (e.g., neuromuscular blocking agents, sedation, and analgesia)^67^. Patients may also have received renal replacement therapy (i.e., intermittent hemodialysis or continuous veno-venous hemofiltration) or underwent venovenous extracorporeal membrane oxygenation, as indicated.

### Blood sample collection

Subjects underwent phlebotomy on the day of enrollment. Venous blood (8.5 mL) was collected from COVID-19 and control subjects using BD Vacutainer ACD tubes. Blood aliquots (50 μL) for flow cytometric analysis underwent erythrocyte lysis with ammonium chloride lysing solution (150 mmol/L NH_4_Cl, 10 mmol/L NaHCO_3_, and 1 mmol/L EDTA, pH 7.4). Blood smears were prepared and fixed in cold 100% methanol; slides were stored at − 20 °C until shipment on dry ice for third-party NETosis analysis.

Platelet-free plasma (PFP) was prepared at room temperature using 2-step centrifugation, each at 2000×*g* for 20 min. After processing, plasma was stored at − 80 °C until further analysis. No more than one freeze/thaw cycle was allowed for PFP samples to prevent protein degradation.

### Flow cytometric analysis

After red blood cell lysis, white blood cells (WBC, 10^6^ cells/mL) were treated with whole molecule mouse and human IgGs to prevent nonspecific binding, followed by incubation with relevant antibodies for 25 min at 4 °C. Cells were washed once with ten volumes of cold PBS/0.5% BSA/2 mM EDTA before data acquisition.

Subpopulations of WBC were analyzed using the following antibodies: CD3 (UCHT1), CD4 (OKT4), CD8a (HIT8a), CD10 (HI10a), CD14 (HCD14), CD16 (3G8), CD26 (BA5b), CD38 (HIT2), CD45 (HI30), CD73 (AD2), HLA-DR (LN2430) (all purchased from BioLegend). Human anti-human/mouse DEspR antibody (NCTX-01) was provided by NControl Therapeutics (Medfield, MA). Human IgG4-S228P isotype control was obtained from Syd Labs (Southborough, MA). Both anti-DEspR and isotype IgGs were conjugated with Alexa Fluor 647 using Molecular Probes^®^ Alexa Fluor^®^ Antibody Labeling Kits (ThermoFisher Scientific). Subpopulations of cells were defined as follows: side scatter (SSC)^high^/CD16^high^/CD14^low/−^/HLA-DR negative neutrophils, SSC^intermediate^/CD14^high^/HLA-DR positive monocytes, SSC^low^/CD14^negative^ lymphocytes. Subsets of CD4 and CD8 T cells coexpressing CD73 and CD26 were determined within the subpopulation of CD3 positive cells.

The total number of WBC was determined using TruCount™ Tubes (BD Biosciences). Viable and non-viable cells were distinguished using 4′,6-diamidino-2-phenylindole (DAPI) to detect dead nucleated cells. Data acquisition was performed on a MacsQuant Analyzer 10 (Miltenyi Biotec, Inc), and the data were analyzed using WinList 5.0 and FlowJo 10.7 software.

### Analysis of circulating IL-6, IL-8, MCP-1, CCL4, CCL23, CXCL2, and CD73

Plasma levels of IL-6, IL-8, MCP-1, CCL4, CCL23, CXCL2, and soluble CD73 were measured using ELISA kits (Bio-techne/R & D Systems).

### Fixed cell imaging of blood smears (NETosing quantification)

Immunofluorescence imaging was performed as a contract research service at Nikon Imaging Laboratory (Cambridge, MA) as follows. Slides were imaged with a Nikon Ti2-E Widefield microscope equipped with a Plan Apo λ 20× objective and Spectra LED light source and controlled by NIS-Elements. An automated JOBS routine in NIS-Elements was used to image 100 evenly spaced positions along with an entire slide. At each position, automatic focus adjustment with the Perfect Focus System (PFS) was followed by sequential imaging with the 395 (Blue), 470 (Green), and 555 (Red) nm LED light to detect DAPI (nuclei), Alexa Fluor 488 (CD11b) and Alexa Fluor 568 (DEspR, hu6g8), respectively. A General Analysis 3 algorithm was used to process image stacks to segment the nuclei, measure circularity, and quantify signal intensity. Generated CSV files were imported to Excel for scoring. Cells were separated into non-NETosing (Circularity > 0.8) and NETosing (Circularity < 0.8) groups. Scoring of CD11b and DEspR expression was based on the mean signal intensities of the respective fluorophore stainings relative to background fluorescence. Boolean operations were used to count the number of cells in each subgroup as graded by the three markers (Yes/No NETosis, +/− CD11b, +/− DEspR).

### Statistical analysis

Data in this study are expressed as mean value and standard error for normal distribution or as median and interquartile range if the distribution is skewed. Comparisons between two groups were performed using Mann–Whitney tests. Comparisons between more than two groups were performed using Kruskal–Wallis test with Dunn’s multiple-comparisons test, or two-way ANOVA with Tukey multiple comparisons test. Correlation analysis was performed using a Spearman (skewed distribution) correlation. ClustVis v2.0 was used to compute hierarchical clustering and heat map on measured clinical and laboratory parameters^68^. *P* < 0.05 was considered significant.
